# University academics’ perceptions regarding the future use of telepresence robots to enhance virtual transnational education: an exploratory investigation in a developing country

**DOI:** 10.1186/s40561-021-00173-8

**Published:** 2021-11-02

**Authors:** Hanaa Ouda Khadri

**Affiliations:** Faculty of Education, Ain Shams University, Cairo, Egypt

**Keywords:** Telepresence robots, Virtual transnational education, Academics’ perceptions, Virtual students’ mobility, Robotic telepresence

## Abstract

There is a growing interest in employing telepresence robots in different educational contexts due to their great potentials to enhance and improve educational experiences for remote learners. However, there is little use of telepresence robots in virtual transnational education contexts in developing countries. There is a lack of knowledge about university academics’ perceptions regarding the future use of telepresence robots to enhance virtual transnational education contexts. This exploratory research aims to fill this gap by seeking to have evidence-based information on whether telepresence robots as a new emerging technology is perceived among university academics as a useful tool for enhancing virtual transnational education or not. This study also seeks to better understand university academics’ perceptions of obstacles that may hinder them from using telepresence robots and the support that they will need. A sample of 46 Egyptian university academics participated in this study. The results of the study revealed that the academics perceived telepresence robots to have a great potential to enhance virtual transnational education. The academics also perceived telepresence robotics to have positive effects on educational activities. Furthermore, the participants indicated a number of barriers regarding integrating telepresence robotics into the teaching and learning activities and identified the types of support they will need. The uptake of telepresence robots to enhance virtual transnational education could lead to a larger international learner population, better virtual transnational education, and more university revenue. Recommendations for future research are also presented.

**Supplementary Information:**

The online version contains supplementary material available at 10.1186/s40561-021-00173-8.

## Introduction

### Conceptualizing virtual internationalization of higher education

Historically, the academic mobility of students and academic staff formed the basis of the internationalization of higher education with the goal of enhancing their quality of teaching, learning, knowledge, and research through cooperation among different academic institutions, cultures, and nations. Today, the internationalization of higher education involves numerous and various actors, objectives, and mechanisms which have become a broader socio-cultural, economic and academic collaboration and competition between or among nations, institutions, and regions. Broadly speaking, internationalization has become a policy-driven process and strategy of the movements of academics, students, socio-academic cultures, academic programs and institutions, academic and institutional systems, and knowledge across academic cultures and political frontiers (de Wit, 2002; Knight, 2008).

Despite the fact that internationalization has become a prevailing term in the twenty-first century higher education institution worldwide, the meaning and concept of internationalization remain ambiguous. The term “internationalization” is formed from one prefix, one word, and one suffix (inter -, nation (al)- and -ization). “Inter” originated from a Latin word that means “between or mutual.” “Nation” means a political system, or a country with a distinct culture. There are many terms used which refer to the internationalization of higher education such as academic mobility, international academic cooperation, study abroad, and international exchange. Concerning curricula, many terms have been used in reference to the internationalization of higher education. Those terms include aspects such as multicultural education, intercultural education, cross-cultural education, education for international understanding, peace education, global education, international studies, etc. (De Wit, 2002).

Currently, the global landscape in internationalization of higher education is challenging and demands innovative ways from service providers to tackle the consequences of the COVID-19 crisis. Many international students all over the world cannot travel to the host country to continue their international education due to the COVID-19 crisis restrictions and other social and financial reasons. Virtual transnational education or virtual mobility is a solution that can be provided. Recently, virtual transnational education has been greatly influenced by the recent developments of smart technologies. In the past, the internationalization of higher education was about traditional practices such as student mobility, curriculum change, staff mobility, and collaboration among or between institutions for developing teaching process and research. Recently, the internationalization of higher education has changed in a very distinct way since its nature has completely changed from the necessity of the physical mobility of students to virtual mobility through developing certain educational activities on online complement or substitute for physical mobility (Knight, 2012).

#### Different interpretations of virtual transnational education

Virtual transnational education, virtual mobility, or collaborative online international learning are three concepts that are used interchangeably because they reflect the interconnection between internationalization and digitalization, and they have become the prominent examples of higher education crossing national borders (Knight, 2016, p. 328). Virtual transnational education has various forms of virtual mobility, and can be approached from different perspectives. There are two forms of virtual mobility: 1- teacher virtual mobility and 2- student virtual mobility, and every form has its own main relevant features (Creelman & Löwe, 2019). The virtual mobility concept was first defined by Bunt-Kokhuis (2001) who created a rather interesting specific definition of virtual mobility where it was described as “the collaborative communication between a faculty member and his/her counterpart(s) mediated by a computer. More often, these meetings will be interactive and take place across national borders and across time zones” (Bunt-Kokhuis 2001, p. 1). Boaretto and Volungevičienė (2013) defined “virtual internationalization” or “virtual transnational education” as the transnational activities that are supported by information and communication technology and organized at the institutional level to make international, collaborative experiences in an educational context possible (Boaretto & Volungevičienė, 2013, p. 7).

It can be argued that virtual transnational education means studying and collaborating over long distances through virtual methods without being physically present. Van de Wende (1997) hold the view that virtual transnational education is an emergent form of internationalization where students interact with their counterparts and educators in other countries through information and communication technologies (ICT), and follow courses offered by institutions abroad. O'Mahony (2014) defined virtual transnational education as “award- or credit-bearing learning undertaken by students who are based in a different country from that of the awarding institution” (p. 8). For the purposes of this study, the author defines virtual transnational education as a form of education that enables international students from different backgrounds and cultures to learn, research, communicate, collaborate, share knowledge and enhance intercultural understanding through creating an educational environment which is supported by implementing smart information and communication technologies to offer the students the same opportunities and benefits as they would have with physical mobility but while they are at their homes and without travelling to the host country.

### Telepresence robots in virtual transnational education: concept, rationale and importance

There is a significant need for tools to keep remote international students connected to the classroom. Remote learners are susceptible to failing to benefit from peer-mediated learning and active social interactions (Corsby & Bryant, 2020; Kreijns et al., 2002). There are numerous studies that investigated the general positive impacts of educational robotics on enhancing academic, communication and social skills of students and offering them impactful learning experiences to enhance their interest, learning, engagement, and academic achievement at various education levels (e.g., Anwar et al., 2019; Barker & Ansorge, 2007; Benitti, 2012; Corsby & Bryant, 2020; Gonnot et al., 2019; Kory et al., 2013; Petre & Price, 2004; Rubenstein et al., 2015; Zhang et al., 2018). Furthermore, robots can enhance collaborative learning where they can incorporate the social dimension in the learning process (Gonnot et al., 2019; Reis et al., 2019; Rosenberg-Kima et al., 2020).

Telepresence robots hold great potentials to make virtual transnational education more immersive through their abilities to allow students to communicate with and navigate through remote educational environments. During the last decade, telepresence robots have gained more attention and widespread popularity in university classrooms, due to the need of teaching staff to find new technologies and methods that enable them to better connect with distance education students (Corsby & Bryant, 2020; Kennedy, 2016; Zhang et al., 2018).

Lister (2020) defines robotic telepresence as the system that “provides the ability for a remote access participant to see and be seen, to hear and be heard, and to move a self-representing mechanism freely in a given space in order to foster engagement and social interactions” (p. 2). Furthermore, robotic telepresence as Bell (2017) explains” has demonstrated its power to enhance remote learners' educational experiences in very important ways” (para. 7). Robotic telepresence and a telepresence robot are two concepts that are used interchangeably. A telepresence robot, is one of the service robots, which “sometimes referred to as a mobile remote presence” (Kristoffersson et al., 2013, p. 1). It is a remote-controlled, wheeled system that incorporates video conferencing equipment onto mobile robot devices and can navigate a distant environment (Fitter et al., 2020).

The mobile robotic telepresence phenomenon was first studied by Paulos and Canny’s research on the PRoP system (Paulos & Canny, 1998). Since then, many researchers have explored the potentials of using telepresence robots in various fields and contexts such as museums (Roberts & Arnold, 2012), interpersonal communication (Ogawa et al., 2011), Medicine (Daruwalla et al., 2010), and education (Conti et al., 2017, 2020; Kwon et al., 2010; Liao & Lu, 2018; Tanaka et al., 2014; Tsui et al., 2011). Despite many countries employing robots as a learning tool to enhance education (Alimisis & Kynigos, 2009; Han, 2012) and help students to promote the skills they need for living and working in the digital age (Chalmers, 2013; Gura, 2012) there is still strong doubts about using robots in educational contexts. Some theoretical and qualitative research results have indicated that educators may have resistance towards using robots because using them would not be in line with the way they realize the principles of teaching and learning practices (Ensign, 2017; Karypi, 2018; Khanlari, 2014). However, the significance of this principled mindset, and its connection to academics’ perceptions regarding the use of telepresence robots in virtual transnational education, have not been investigated yet.

Past research has explored telepresence robots for remote students support (Bell, 2017; Gleason & Greenhow, 2017; Liao & Lu, 2018; Reis et al., 2019), but to the best of my knowledge, no past research has examined academics’ perceptions regarding the future use of telepresence robots to enhance virtual transnational education to make it feel a little more like real transnational education. Thus, more exploration is needed to fill this gap in the existing literature. This study aimed to contribute to the current literature in the field of educational robotics by investigating university academics’ perceptions regarding the future use of telepresence robots to enhance virtual transnational education. This study also seeks to better understand university academics’ perceptions of the barriers that may hinder them from using telepresence robots and the support they need. The overall goal of this study is to provide insights that may guide ongoing and future developments in virtual transnational education supported by telepresence robots. The goal of this study can be achieved through answering the following main question:

- *How do university academics perceive the future use of telepresence robots to enhance virtual transnational education?*

### Research questions

The five research questions that have guided the study are:

1. What are the academics’ experiences with smart educational technologies?
2. What are the academics’ proficiency levels regarding using telepresence robots’ technology?
3. What are the potential obstacles that may hinder academics from using telepresence robots to enhance virtual transnational education?
4. What kind of support do academics need in order to uptake telepresence robots in virtual transnational education?
5. What are the academics’ perceptions regarding the potential benefits that may encourage them to employ telepresence robots in virtual transnational education to enhance it?

## Literature review

### Telepresence robots for virtual transnational education

In 1980 Marvin Minsky coined the term telepresence for the first time in the context of teleoperation to describe physical labor from remote locations (Minsky, 1980). Recently, telepresence is used to describe a human’s presence in a wide variety of virtual environments by using a telerobot. An intelligent telepresence robot is a smart video conferencing computer with a microphone and smart speakers attached. It can sit on desks or stand in the classroom or even move around. This technology has become progressively prevailing in the education sector during the pandemic, thanks to new viable virtual and hybrid learning models, where some students are in the onsite classroom while remote students watch from home. The big difference between telepresence robots and conventional cameras is that the telepresence robots follow actions and sounds—spinning in a wide 360-degree angle—to give international students a more natural classroom experience. So, students at their home country can see more than static shots of the classroom. Telepresence robots can empower students to contribute to classroom discussions, move to different areas of the classroom to engage in group work (Ahumada-Newhart & Eccles, 2020; Ahumada-Newhart & Olson, 2019; Zhang et al., 2018). There are a wide variety of commercially flexible telepresence robots that could be used for many purposes in many contexts including: hospitals, elderly care, offices, and education. Some of the existing commercial robots are: “BotEyes – Pad, PadBot P1, PadBot U1- Version 2, BotEyes Mini, … Kubi, Ohmni SuperCam, Ohmni, Ava 500, Amy A, Double 3 and many more.” (Yatagiri, 2020)

The uptake of telepresence robots allows remote international students to truly have a seat at the classroom in the host country, both physically and figuratively speaking. Furthermore, it offers distant students the opportunity to engage in real-time class community and conversation, thus they get better learning experiences (Kennedy, 2016; Liao & Lu, 2018). Many universities such as Oral Roberts University and Florida International University are using telepresence robots to better engage their students no matter where they are in real-time class community and conversation (Kennedy, 2016; Reis et al., 2019).

Bell (2017) argued that telepresence robots are game-changers due to their potentials to enhance and improve educational experiences for remote learners and has made the teaching and learning strategies much easier (Kennedy, 2016; Liao & Lu, 2018). Moreover, it supports collaborative learning (Reis et al., 2019; Rosenberg-Kima et al., 2020). Telepresence robots have various benefits and challenges. It is of great importance to consider pragmatic issues when selecting them. These issues include selecting the best fit telepresence robot in terms of price, required networks and bandwidth. In addition, confidence of use, freedom to move around, great social presence and support for various types for engagement are very critical variables guiding the process of selection (Bell, 2017; Yousif, 2021; Zhang et al., 2018). Moreover, universities should have flawlessly working wireless technology (Kennedy, 2016; Yousif, 2021).

## Methodology and procedures

The goal of this exploratory study is to explore the perceptions of university academics regarding the future use of telepresence robots to enhance virtual transnational education. This study is qualitative in nature, due to its purpose; that is, how university academics perceive the future use of telepresence robots to enhance virtual transnational education. As Merriam (1998) stated, “[q]ualitative research is a journey of discovery rather than confirmation” (p. 18) in which researchers can implement various methodologies to gain in-depth understanding of others’ thoughts and experiences. In line with this, Henderson (1991) stated that qualitative research empowers researchers to gain insight into others’ attitudes, perceptions, beliefs, and opinions.

### Tool of data collection

As the participants may have a little or no experience in the uptake of telepresence robots as a teaching and learning tool, the participants were provided relevant links, websites and information about using telepresence robots as educational tools. The participants reviewed the links, websites and information before the interview sessions or filling in the online surveys. A semi-standardized, open-ended interview was developed. The interview included 19 questions. An online survey was created and sent to the participants. The questionnaire has been prepared after reviewing the related literature. The questionnaire included four sections: (1) demographic and professional characteristics, (2) experience with smart educational technologies, (3) using telepresence robots, (4) and academics’ overall perceptions of telepresence robots. It is noteworthy, all the participants have preferred to fill in the survey rather than to be interviewed face to face, because most of them are concerned about that they may be infected with the COVID-19 virus and they are very busy. To guarantee anonymity, universities and participants responses were numbered (e.g., UNI 1, participant 1).

The Cronbach's alpha coefficient for the questionnaire has been measured to determine its internal consistency of each section and its items. It ranged between (0.82–0.92) while the overall stability of it reached (0.90), as shown in Table 1, which are highly stable coefficients and meets the purposes of the study. Questionnaire face validity was assured after the revision conducted by eight academic experts and some modifications have been done according to their opinions. The questionnaire was piloted to seven of teaching staff and it was revised according to the pilot test results.

**Table 1:** Cronbach’s alpha of the questionnaire and its sections

Total stability		Section one		Section two		Section three		Section four	
Stability coefficient	Number of items	Stability coefficient	Number of items	Stability coefficient	Number of items	Stability coefficient	Number of items	Stability coefficient	Number of items
0.90	20	0.92	9	0.90	3	0.82	6	0.89	1

### Target group and participants

The participants of this study included any academics in public Egyptian universities. The researcher, through the use of the purposive sampling technique, selected academics that represented different fields of specialization from five public Egyptian universities that recently established new faculties or departments of Artificial Intelligence (Ain Shams University, Mansoura University, Zagazig University, Al Azhar University, Helwan University) given also the very limited number of academics who could be involved in educational robots’ projects in Egypt. The data were collected during the government implementation of preventive measures in Egypt (March–April 2020). The sample consists of 46 academics from five public Egyptian universities.

The selected participants were contacted to request their participation in this study. The participants were directed to links about the topic of the study, the survey (see Additional file 1). Although the participants had three options to either participate in telephone or face-to-face interviews or complete an online survey, all 46 participants preferred to participate in the study by filling in the online survey. Moreover, telephonic calls discussion was also carried out by the researcher with participants to discuss the research topic and its objectives.

### Statistical analyses

Descriptive statistics were employed, and SPSS version 23.0 was utilized. The data collected indicated several significant information about academics’ perceptions regarding the future use of telepresence robots in educational activities to enhance virtual transnational education. The data has been summarized into tables. Descriptive statistics (frequencies, percentages, means and standard deviations) were calculated for the question.

## Results

This exploratory study focused on university academics’ perceptions regarding the future use of telepresence robots to enhance virtual transnational education. In order to achieve the study objectives, the author used the online questionnaire to collect the required data.

### Questionnaire section 1: demographic and professional characteristics

Section 1 aimed at describing the participants’ demographic and professional characteristics (i.e., gender, age, faculty rank, years of teaching experience, years of using technology in teaching, and preferred teaching methodology) and students’ and academics’ access to smart educational technologies. Participants’ demographic and professional characteristics are summarized in Table 2.

**Table 2:** Summary of participants’ demographic and professional characteristics

Variable	Values	N	% (of total)
Gender	Male	25	54.35
	Female	21	45.65
Age	> 50 years	17	36.96
	40–50 years	13	28.26
	< 40 years	16	34.78
Faculty rank	Lecturer	16	34.78
	Associate Professor	19	41.30
	Professor	11	23.91
Faculty	Engineering	10	21.47
	Medicine	9	19.57
	Computer Science	9	19.57
	Education	10	21.47
	Science	8	17.39
Years of university teaching experience	> 10 years	11	23.91
	5–10 years	19	41.30
	< 5 years	16	34.78
Years of using technology in teaching	> 10 years	0	0
	5–10 years	15	32.61
	< 5 years	31	67.39
Preferred teaching methodology	Flexible teaching activities that (combination of teacher-directed and platform-directed instruction)	7	15.22
	Mixture of teacher-centered and student-centered activities	31	67.39
	Teacher-directed activities	3	6.52
	Student-centered activities	5	10.87

As regards to the participants’ demographic and professional characteristics, it has been observed that the sample was evenly distributed concerning their faculties (Engineering, Medicine, Computer Science, Education, Science). The participants are predominantly male (*n* = 25, 54. 35%) and are mostly either associate professors (41.30%) or lecturer (34.78%). The mean age of the participants was 47.70 years (*SD* = 13.628), and the faculty rank was consistent with the faculty age. The average sample of teaching experience was 1.89 years (range 4 to 35 years of experience). Finally, the average sample of using technology in teaching was 1.35 years (range 1 to 10 years of experience).

It has been observed that the majority of the participants (67.39%) preferred mixture of teacher-centered and student-centered activities, three participants (6.52%) stated that they prefer teacher-directed activities, five participants (10.87%) preferred student-centered activities, and seven participants (15.22%) preferred flexible teaching activities (combination of teacher-directed and platform-directed instruction). Moreover, participants were asked to rate students and academics access to smart educational technologies at their university. The results showed that all participants rated access to smart educational technologies to be above 67% as shown in Table 3. These results were expected due to the great efforts of the Egyptian Ministry of Higher Education and Scientific Research in collaboration with the Minister of Communications and Information Technology to expand students and academics’ access to smart educational technologies to make best use of the advanced technological systems in Egyptian universities to build digital Egypt (El Said, 2021).

**Table 3:** Students and academics’ access rate to smart educational technologies

Participant	Students’ access to smart educational technologies (%)	Academics’ access to smart educational technologies (%)	Participant	Students’ access to smart educational technologies (%)	Academics’ access to smart educational technologies (%)
Participant 1	69	79	Participant 24	68	77
Participant 2	79	86	Participant 25	76	84
Participant 3	75	78	Participant 26	77	81
Participant 4	72	79	Participant 27	72	79
Participant 5	90	95	Participant 28	90	95
Participant 6	67	71	Participant 29	67	71
Participant 7	89	94	Participant 30	90	92
Participant 8	75	78	Participant 31	85	88
Participant 9	91	95	Participant 32	85	91
Participant 10	79	87	Participant 33	77	80
Participant 11	84	91	Participant 34	83	89
Participant 12	80	87	Participant 35	70	75
Participant 13	68	73	Participant 36	67	70
Participant 14	75	85	Participant 37	85	89
Participant 15	81	90	Participant 38	91	95
Participant 16	85	91	Participant 39	75	82
Participant 17	87	92	Participant 40	84	92
Participant 18	90	94	Participant 41	76	84
Participant 19	83	86	Participant 42	73	76
Participant 20	79	83	Participant 43	89	93
Participant 21	75	81	Participant 44	75	82
Participant 22	67	72	Participant 45	87	92
Participant 23	91	93	Participant 46	77	83

### Questionnaire section 2: Academics’ experience with smart educational technologies

This section listed three different questions. The first question (Question 10) aimed at identifying the types of smart technologies that academics use most in their teaching and learning activities by asking participants “*If you use smart educational technologies in your educational activities, what type of smart technologies you use most?*” Table 4 summarizes academics’ responses to this question.

**Table 4:** Smart educational technologies used by academics

Participant	Smart educational technologies	Participant	Smart educational technologies
Participant 1	Interactive whiteboards, smartphones computers, websites, computer lab	Participant 24	Computers, smartphones, smart boards, software programs, websites
Participant 2	Computers, interactive whiteboards, Laptops, computer lab, virtual reality labs, software programs	Participant 25	Computer lab, Internet, smart boards, software programs, Laptops, smartphones
Participant 3	Computers, websites, smartphones, smart boards, software programs	Participant 26	Computer lab, smart boards, Internet, laptops
Participant 4	software programs, computers, smartphones, websites, computer lab, smart boards	Participant 27	Websites, smart boards, software programs, smartphones, computer lab
Participant 5	Computers, software programs, iPads, Websites, Educational Games, interactive whiteboards, virtual reality, cloud computing, learning analytics	Participant 28	Laptops, iPads, computer lab, interactive whiteboards, virtual reality, software programs
Participant 6	Smart boards, computers, smartphones, websites, software programs, internet	Participant 29	Smart boards, Internet, smartphones, laptops computer lab, software programs
Participant 7	Computers, software programs, iPads, virtual reality, websites, interactive whiteboards	Participant 30	Computers, interactive whiteboards, software programs, iPads, Websites, virtual reality labs
Participant 8	Smart boards, websites, smartphones, software programs, computer lab, smartphones, internet	Participant 31	Websites, smartphones, computer lab, software programs, interactive whiteboards, virtual reality
Participant 9	Laptops, software programs, iPads, blogs, computer lab, wikis, educational games, interactive whiteboards, virtual reality, cloud computing	Participant 32	Computers, software programs, iPads, Websites, Educational Games, interactive whiteboards, virtual reality, cloud computing, learning analytics
Participant 10	Computers, interactive whiteboards, smartphones, laptops, computer labs, software programs	Participant 33	Software programs, iPads, Websites, interactive whiteboards, virtual labs
Participant 11	Computer lab, interactive whiteboards, virtual reality, software programs, iPads, websites, cloud computing	Participant 34	Virtual labs, computers, iPads, websites, smartphones, interactive whiteboards
Participant 12	Computers, smart boards, Laptops, computer labs, software programs, websites, Google docs,	Participant 35	Smart boards, websites, computer lab, virtual reality
Participant 13	Computer lab, smartphones, smart boards, websites	Participant 36	Smart boards, virtual reality, websites, software programs, computer lab
Participant 14	Smart boards, websites, software programs, computer lab, internet	Participant 37	Software programs, interactive whiteboards, laptops, computer lab, virtual reality
Participant 15	Computer labs, smartphones, websites, smart boards	Participant 38	Interactive whiteboards, software programs, iPads, laptops, discussion forums, computer lab, educational games, virtual reality, mixed reality (MR)
Participant 16	Computer lab, interactive whiteboards, virtual labs, software programs, iPads, websites, discussion forums,	Participant 39	Virtual reality, software programs, interactive whiteboards, computer lab,
Participant 17	Interactive whiteboards, virtual labs, software programs, iPads, websites	Participant 40	Smart boards, virtual reality labs, computer lab, software programs, iPads
Participant 18	Computers, software programs, iPads, Websites, Educational Games, interactive whiteboards, virtual reality	Participant 41	Interactive whiteboards, smartphones, virtual reality, websites
Participant 19	Interactive whiteboards, virtual labs, software programs, iPads, websites	Participant 42	Interactive whiteboards, virtual labs, software programs, iPads, websites
Participant 20	Interactive whiteboards, virtual labs, software programs, iPads, websites	Participant 43	Smart boards, virtual reality websites, computer lab, software programs, iPads
Participant 21	Interactive whiteboards, Laptops, smartphones, computer lab, websites	Participant 44	Smart boards, smartphones, educational games, virtual reality, websites
Participant 22	Computers, Smart boards, software programs, websites	Participant 45	Virtual labs, software programs, iPads, websites, interactive whiteboards
Participant 23	Smart boards, iPads, software programs, discussion forums, Laptops, computer lab, virtual reality	Participant 46	Computers, smartphones, interactive whiteboards, computer labs, websites

Question 11 aimed at identifying the frequency academics use various smart educational technologies in their educational activities. To define how often the participants use various smart educational technologies in their educational activities, the following six-point response scale was used: 0 = Never, 1 = Rarely, 2 = Occasionally, 3 = Frequently, 4 = Almost Always, and 5 = All the time.

Twelve academics (26.1%) frequently use various smart educational technologies in their educational activities, eleven academics (23.8%) stated that they rarely use various smart educational technologies, five academics (10.9%) identified that they almost always use smart educational technologies, nine academics (19.6%) occasionally use various smart educational technologies, nine academics (19.6%) all the time use various smart educational technologies, and no academics (0%) stated that they never used smart educational technologies in their educational activities (Table 5) which represents frequencies and percentages for each variable ordered by the highest value.

**Table 5:** Academics’ responses regarding the frequency they use various smart educational technologies in their educational activities

Variable	N	% (of total)
Frequently	12	26.1
Rarely	11	23.8
Almost always	5	10.9
Occasionally	9	19.6
All the time	9	19.6
Never	0	0
	46	100

Participants were also asked to select the proficiency level that best describe them as users of telepresence robots. In response to the question, twenty-four academics (52.17%) stated that they are unfamiliar with telepresence robots and have no experience in working with robotics technology, while five academics (10.87%) stated that they are newcomers, and seven academics (15.22%) are beginners, and seven academics (8.70%) stated that they have ordinary competency level, three academics (6.52%) have advanced proficiency level (6.52%), and three academics (6.52%) have expert proficiency level. This finding is expected, as telepresence robots are new as an educational technology in developing countries (Ponce et al., 2019) (Table 6).

**Table 6:** Academics’ proficiency levels regarding using telepresence robots’ technology

Proficiency levels	Number and percentage (%)
*Unfamiliar* I do not have experience with telepresence robots’ technology	24 (52.17%)
*Newcomer* I have tried to employ telepresence robots’ technology, but I still need help regularly	5 (10.87%)
*Beginner* I can carry out core functions in a few numbers of telepresence robots’ applications	7 (15.22%)
*Average* I show an ordinary competency in some telepresence robots’ applications	4 (8.70%)
*Advanced* I have the ability to competently employ a diverse range of telepresence robots’ applications	3 (6.52%)
*Expert* I am highly competent in employing telepresence robots’ applications	3 (6.52%)

### Questionnaire section 3: using telepresence robots

Academics were also asked to determine their experience with using telepresence robots in their educational activities. While, forty-one (89.13%) of the academics mentioned that they have never employed telepresence robots in their educational activities, only five academics (10.87%) used telepresence robots in their educational activities. Moreover, academics were asked to indicate the total amount of professional development they received to employ telepresence robots in their educational activities, twenty-four academics (52.17%) stated that they have not received any training to employ telepresence robots in their educational activities, while twenty-two academics (47.83%) stated that they had received more than a full day professional development workshop.

Academics were also asked to determine the total amount of professional development they need to employ telepresence robots in their educational activities. Three academics (6.5%) stated that they need a full day or less professional development workshop, while twenty-six academics (56.5%) stated that they need more than one-semester professional development programs, five academics (10.9%) stated that they need a one semester course, and twelve academics stated that they need more than a one semester course (Table 7).

**Table 7:** Academics’ perceptions of the total amount of professional development they need

Variable	N	% (of total)
A full day or less	3	6.5
More than a full day	26	56.5
A one semester course	5	10.9
More than a one semester course	12	26.1

Question 16 aimed at identifying the stage of the process of employing telepresence robots into educational activities. Sixteen academics (34.78%) stated that they are aware of the existence of the telepresence robot, but never used it, However, seven academics (15.22%) stated that they are attempting to learn the core basics of using telepresence robots, and three academics (6.52%) stated that they have begun to understand how to employ telepresence robots and can suggest some applications in which they might be effective (Table 8).

**Table 8:** Six stages of the process of employing telepresence robots into educational activities

Stages’ descriptions	Number and percentage (%)
*Awareness* I am aware of the existence of the telepresence robot, but never used it. I am worried about the expectations of employing telepresence robots	16 (34.78%)
*Learning* I am attempting to learn the core basics of using telepresence robots. I am often dissatisfied about employing telepresence robots and I do not have confidence when using them	7 (15.22%)
*Understanding* I have begun to understand how to employ telepresence robots and can suggest some applications in which they might be effective	3 (6.52%)
*Familiarity* I am building self-confidence in employing telepresence robots for certain educational activities. I feel comfortable to some extent when employing the telepresence robots	0 (0%)
*Adaptation* I think telepresence robots are educational tools that can enable me in carrying out some educational practices and I am not anxious about them as technologies. I can employ a wide range of telepresence robots’ applications	0 (0%)
*Creative application* I have the ability to apply all I know about telepresence robots in different education contexts. I can use them as instructional tools and have integrated them into the courses I teach	0 (0%)

Question 17 aimed to explore academics’ perceptions of the potential obstacles that may hinder them from using telepresence robots in virtual transnational education as: no obstacle at all, minor obstacle, moderate obstacle, severe obstacle, or a very severe obstacle. Table 9 shows academics’ responses.

**Table 9:** Potential obstacles that may hinder academics from using telepresence robots

Potential obstacles	% Academics identified as			
	Minor obstacle	Moderate obstacle	Severe obstacle	Very severe obstacle
*PO1* Using telepresence robots would not be in line with the way I realize the principles of teaching and learning practices	1 (2.2%)	39 (84.8%)	3 (6.5%)	3 (6.5%)
*PO2* The difficulty of knowing how to implement the telepresence robot system architecture	19 (41.3%)	21 (45.7%)	3 (6.5%)	3 (6.5%)
*PO3* There are not enough number of telepresence robots available in university	0 (0%)	0 (0%)	0 (0%)	46 (100%)
*PO4* Generally, academics do not have access to the related software	0 (0%)	0 (0%)	0 (0%)	46 (100%)
*PO5* Usually, there are not enough computers for programing the telepresence robots	0 (0%)	0 (0%)	0 (0%)	46 (100%)
*PO6* Using telepresence robots require more effort and time for classroom management	0 (0%)	0 (0%)	0 (0%)	46 (100%)
*PO7* using telepresence robots will increase the amount of stress for academics because some international students may know more about telepresence robotics than some academics do	0 (0%)	0 (0%)	0 (0%)	46 (100%)
*PO8* Academics do not feel confident enough to use telepresence robots in their educational activities	0 (0%)	0 (0%)	0 (0%)	46 (100%)
*PO9* Lack of sufficient administrative support	0 (0%)	0 (0%)	0 (0%)	46 (100%)
*PO10* lack of appropriate technological support	0 (0%)	0 (0%)	0 (0%)	46 (100%)
*PO11* Lack of reference cases	0 (0%)	0 (0%)	0 (0%)	46 (100%)
*PO12* The difficulty of the integration of telepresence robots in a classroom environment	0 (0%)	0 (0%)	0 (0%)	46 (100%)

All the participants stated that all the mentioned obstacles are very severe, except for PO1 and PO2 which most of the participants considered them as moderate ones.

Table 10 shows academics’ responses to question 18, which was: “What kinds of support that academics need in order to uptake telepresence robots in virtual transnational education?”

**Table 10:** Kinds of support that academics need in order to use telepresence robots

Kinds of support	Number and percentage (%)
How to select the best fit telepresence robot	41 (89.1%)
Appropriate logistical support	37 (80.4%)
Technological support regarding knowing how to implement the telepresence robot system architecture	46 (100%)
Pre-service and in-service appropriate capacity building	46 (100%)
Pedagogical support regarding the educational collaborative activities and assessment procedures that should be adapted to the use of telepresence robots	38 (82.6%)
How to align educational environments with the requirements of using telepresence robots to maximize learners’ potentials	38 (82.6%)
Building confidence in using telepresence robots among academics and stakeholders	43 (93.5%)

### Questionnaire section 4: academics overall perceptions

As regards to academics’ responses to question 19, which was a 5-point Likert Scale question regarding the potential benefits that may encourage academies to employ telepresence robots in virtual transnational education.

Table 11 presents means and standard deviations for the academics’ responses regarding each potential benefit of employing telepresence robots in virtual transnational education ordered by the highest mean value. Higher mean values indicate a higher level of agreement between academics on the potential benefits, whereas lower mean values indicate a lower level of agreement. As shown in Table 11, it turns out to be relatively high (M = 4.67, SD = 0 0.442).

**Table 11:** Academics’ responses regarding the potential benefits of employing telepresence robots in virtual transnational education

Potential benefits	% Academics identified as						
	Strongly disagree	Disagree	Neutral	Agree	Strongly agree	M	SD
*Pb1* Make virtual international education feel a little more like face-to-face transnational education	0	0	0	3 (6.5%)	34 (93.5%)	4.93	0.250
*Pb2* Help remote students to stay connected to educators and other students	0	0	0	3 (6.5%)	34 (93.5%)	4.93	0.250
*Pb3* Enhance and improve educational experiences for remote learners	0	0	0	8 (17.4%)	38 (82.6%)	4.83	0.383
*Pb4* Promote collaborative learning in an authentic and interactive environment	0	0	0	8 (17.4%)	38 (82.6%)	4.83	0.383
*Pb5* Enable remote international students to exchange their ideas and opinions with their peers	0	0	0	9 (19.6%)	37 (80.4%)	4.80	0.401
*Pb6* Develop positive attitude about virtual transnational education	0	0	0	9 (19.6%)	37 (80.4%)	4.80	0.401
*Pb7* Help students to gain the skills they need for living and working in the digital age	0	0	0	9 (19.6%)	37 (80.4%)	4.80	0.401
*Pb8* Encourage students to pursue their international education	0	0	0	11 (23.94%)	35 (76.1%)	4.76	0.431
*Pb9* Can tackle the consequences of the COVID-19 crisis on international students	0	0	0	13 (28.3%)	33 (71.7%)	4.72	0.455
*Pb10* Improve technology literacy of academics and students	0	0	0	21 (45.7%)	25 (54.3%)	4.54	0.504
*Pb11* Empower international students to learn with their peers, despite geographic distance	0	0	1 (2.2%)	22 (47.8%)	23 (50.0%)	4.48	0.547
*Pb12* Can tackle the challenges of virtual internationalism of higher education	0	0	3 (6.5%)	21 (45.7%)	22 (47.8%)	4.41	0.617
*Pb13* facilitate teaching and learning activities	0	0	3 (6.5%)	27 (58.7%)	16 (34.8%)	4.28	0.584
*Pb14* Offer new opportunities for academics to be learning facilitators rather than knowledge providers	0	0	3 (6.5%)	27 (58.7%)	16 (34.8%)	4.28	0.584
Potential benefits						4.67	0.442

## Conclusions, discussion and future work

The idea of virtual transnational education has gained great prominence as a consequence of the COVID-19 pandemic. Virtual transnational education is rooted in the notion of enabling students to study, collaborate with educators and peers from other countries through using the up-to-date information and communication technologies. Telepresence robots are remarkably innovative educational tools that can enhance virtual transnational education to make it feel a little more like a real one. The results of this study indicate that university academics perceive the important educational potentials of telepresence robots. Therefore, this study concurs with Kwon et al. (2010), Ogawa et al. (2011), Benitti (2012), Kristoffersson et al. (2013), Tanaka et al. (2014), Rubenstein et al. (2015), Kennedy (2016), Bell (2017), Conti et al. (2017), Anwar et al. (2019), Gonnot et al. (2019), Lister (2020), Fitter et al. (2020), Corsby and Bryant (2020) and Rosenberg-Kima et al. (2020) regarding the positive effects of robots on enhancing educational activities for remote students. Specifically, the study results concur with Bell (2017), Gleason and Greenhow (2017) and Liao and Lu (2018) regarding that telepresence robots can enhance education for remote students.

Although all the participants stated that their access to smart educational technology is above 67% and they use various smart technologies in their educational activities, 16 (34.78%) academics stated that they have never used telepresence robots in their educational activities. This finding is quite expected, as telepresence robots are new as an educational technology (Cha et al., 2017; Newhart et al., 2016; Yousif, 2021). Despite the above-mentioned educational benefits of telepresence robots, this study revealed that using telepresence robots to enhance virtual transnational education has many potential obstacles that academics may encounter, especially as regards to the use of telepresence robots which would not be in line with the way they realize the principles of teaching and learning practices and they do not feel confident enough to use telepresence robots in their educational activities because they think it will be difficult for them to implement the telepresence robot system architecture. In accordance with the existing literature (Kennedy, 2016; Liao & Lu, 2018; Theodoropoulos et al., 2017; Yousif, 2021), the results showed that the obstacles included inadequate access to the related hardware and software, inadequate administrative and technological support, the difficulty of integration of telepresence robots in a traditional classroom environment, and lack of reference cases and enough knowledge about telepresence robots.

Furthermore, the analysis of the data revealed that all the academics perceived that pre-service and in-service appropriate capacity building, technological support regarding knowing how to implement the telepresence robot system architecture, and building confidence in using telepresence robots among academics and stakeholders are the most important support they need. Most of the academics (56.5%) stated that more than a full day is enough for them to be able to successfully use telepresence robots in their educational activities, and (26.1%) academics perceived that they need more than one semester course. Pedagogical, logistical and technological support are other types of support that academics perceived to be very important. In this respect, to make it possible to use telepresence robots to enhance virtual transnational education, actions should be taken both centrally and at universities’ level. An important action that can make it work in this direction which is carrying out pre-service and in-service appropriate capacity building programs.

Overall, the analysis of the results suggests that there is positive perception of academics regarding the uptake of telepresence robots in a virtual transnational education to enhance it. Finally, an in-depth dialogue should be opened among all the stakeholders on the great importance of the uptake of telepresence robots to enhance virtual transnational education by adopting supportive educational policies and strategies and the best practices and reference cases developed in developed countries. Future research is needed as regards to the institutional factors which influence the integration of telepresence robots in higher education, as well as exploring the students’ perceptions of learning via telepresence robots, in order to better understand telepresence robots’ educational potentials and limitations. Furthermore, no single solution is always best, so it is recommended that future research continue exploring and experimenting with a variety of solutions to figure out what solution is preferable in a particular educational context for a specific goal, since the capabilities of telepresence robots technology are evolving and expanding.

## Supplementary Information

**Additional file 1.** The Manuscript.

## Data Availability

Data sharing is not applicable to this article as no datasets were generated or analyzed during the current study.

## Abbreviation

ICT: Information and Communication Technology
