# Supplementary material for: University academics’ perceptions regarding the future use of telepresence robots to enhance virtual transnational education: an exploratory investigation in a developing country
Source: Smart Learn Environ. 2021 Nov 2;8(1):28. doi: 10.1186/s40561-021-00173-8 (PMC8561341; doi:10.1186/s40561-021-00173-8)
Supplement: Supplementary file 1 — Additional file 1. The Manuscript. [file 40561_2021_173_MOESM1_ESM.docx]

**Appendix A**

**Questionnaire on University Academics’ Perceptions about the Future Use of Telepresence Robots** **to Enhance Virtual Transnational Education: An Exploratory Analysis in a Developing Country**

Section 1: demographic and professional characteristics

1. Gender (optional)

*Please check one answer below*

Male

Female

1. Age

*Please check one answer below*

*>50 years*

*40–50 years*

*<40 years*

1. Name of your university and faculty

*Please type your answer below*

1. Faculty Rank

*Please check one answer below*

Lecturer

Associate Professor

Professor

1. Years of university teaching experience

*Please check one answer below*

>10 years

5–10 years

<5 years

1. How many years have you used technology in teaching?

*Please check one answer below*

>10 years

5–10 years

<5 years

1. What is your preferred teaching methodology?

*Please check one answer below*

Flexible teaching activities (combination of teacher-directed and platform-directed instruction)

Mixture of teacher-centered and student-centered activities

Teacher-directed activities

Student-centered activities

1. How would you rate students’ access to smart educational technologies at your university? (0-100 scale)
2. How would you rate academics’ access to smart educational technologies at your university? (0-100 scale)

Section 2: experience with smart educational technologies

1. If you use smart educational technologies in your educational activities, what type of smart technologies you use most?

Please define briefly in the box below

1. Kindly define how often you use various smart educational technologies in your educational activities?

*Please check one answer below*

Never

Rarely

Occasionally

Frequently

Almost always

All the time

1. Kindly determine the proficiency level that best describe you as a user of telepresence robots.

*Please check one answer below*

Unfamiliar: I do not have experience with telepresence robots’ technology.

Newcomer: I have tried to employ telepresence robots’ technology, but I still need help regularly.

Beginner: I can carry out core functions in a few numbers of telepresence robots’ applications.

Average: I show an ordinary competency in some telepresence robots’ applications.

Advanced: I have the ability to competently employ a diverse range of telepresence robots’ applications.

Expert: I am highly competent in employing telepresence robots’ applications.

Section 3: Using telepresence robots

1. Kindly determine your experience with using telepresence robots in your educational activities.
2. Kindly indicate the total amount of professional development you received to employ telepresence robots in your educational activities.
3. Kindly indicate the total amount of professional development you need to employ telepresence robots in your educational activities.

- A full day or less
- More than a full day
- A one semester course
- More than a one semester course

1. Kindly read the following stages of employing telepresence robots into educational activities. Select the stage that best describe your current performance.

Awareness: I am aware of the existence of the telepresence robot, but never used it. I am worried about the expectations of employing telepresence robots.

Learning: I am attempting to learn the core basics of using telepresence robots. I am often dissatisfied about employing telepresence robots and I do not have confidence when using them.

Understanding: I have begun to understand how to employ telepresence robots and can suggest some applications in which they might be effective.

Familiarity: I am building self-confidence in employing telepresence robots for certain educational activities. I feel comfortable to some extent when employing the telepresence robots.

Adaptation: I think telepresence robots are educational tools that can enable me in carrying out some educational practices and I am not anxious about them as technologies. I can employ a wide range of telepresence robots’ applications.

Creative application: I have the ability to apply all I know about telepresence robots in different education contexts. I can use them as instructional tools and have integrated them into the courses I teach.

1. The following statements could be the potential obstacles that may hinder you from using telepresence robots in virtual transnational education. Please classify each one of them as follows:(minor obstacle -moderate obstacle -severe obstacle -very severe obstacle)?

| Potential obstacles | Minor obstacle | Moderate obstacle | | Severe obstacle | | Very severe obstacle | |
| --- | --- | --- | --- | --- | --- | --- | --- |
| PO1: Using telepresence robots would not be in line with the way I realize the principles of teaching and learning practices. |  | |  | |  | |  |
| PO2: The difficulty of knowing how to implement the telepresence robot system architecture. |  | |  | |  | |  |
| PO3: There are not enough number of telepresence robots available in university. |  | |  | |  | |  |
| PO4: Generally, academics do not have access to the related software. |  | |  | |  | |  |
| PO5: Usually, there are not enough computers for programing the telepresence robots. |  | |  | |  | |  |
| PO6: Using telepresence robots require more effort and time for classroom management. |  | |  | |  | |  |
| PO7: using telepresence robots will increase the amount of stress for academics because some international students may know more about telepresence robotics than some academics do. |  | |  | |  | |  |
| PO8: Academics do not feel confident enough to use telepresence robots in their educational activities. |  | |  | |  | |  |
| PO9: Lack of sufficient administrative support. |  | |  | |  | |  |
| PO10: lack of appropriate technological support. |  | |  | |  | |  |
| PO11: Lack of reference cases. |  | |  | |  | |  |
| PO12: The difficulty of the integration of telepresence robots in a classroom environment. |  | |  | |  | |  |

1. What kinds of support that academics need in order to uptake telepresence robots in virtual transnational education?

Please specify in the box below

Section 4: academics overall perceptions

1. The following statements indicates the potential benefits that may encourage academies to employ telepresence robots in virtual transnational education to enhance it.

Please indicate your response to these statements. (Strongly Disagree, Disagree, Neutral, Agree, or Strongly Agree)

| Potential benefits | Strongly  Disagree | Disagree | Neutral | Agree | Strongly  Agree |
| --- | --- | --- | --- | --- | --- |
| Pb1: Make virtual international education feel a little more like face-to-face transnational education. |  |  |  |  |  |
| Pb2: Help remote students to stay connected to educators and other students. |  |  |  |  |  |
| Pb3: Enhance and improve educational experiences for remote learners. |  |  |  |  |  |
| Pb4: Promote collaborative learning in an authentic and interactive environment. |  |  |  |  |  |
| Pb5: Enable remote international students to exchange their ideas and opinions with their peers. |  |  |  |  |  |
| Pb6: Develop positive attitude about virtual transnational education. |  |  |  |  |  |
| Pb7: Help students to gain the skills they need for living and working in the digital age. |  |  |  |  |  |
| Pb8: Encourage students to pursue their international education. |  |  |  |  |  |
| Pb9: Can tackle the consequences of the COVID-19 crisis on international students. |  |  |  |  |  |
| Pb10: Improve technology literacy of academics and students. |  |  |  |  |  |
| Pb11: Empower international students to learn with their peers, despite geographic distance. |  |  |  |  |  |
| Pb12: Can tackle the challenges of virtual internationalism of higher education. |  |  |  |  |  |
| Pb13: facilitate teaching and learning activities. |  |  |  |  |  |
| Pb14: Offer new opportunities for academics to be learning facilitators rather than knowledge providers. |  |  |  |  |  |
| Potential benefits |  |  |  |  |  |

Thank you for taking the survey.
